# Octopus‐Inspired Adhesives with Switchable Attachment to Challenging Underwater Surfaces

**DOI:** 10.1002/advs.202407588

**Published:** 2024-10-09

**Authors:** Chanhong Lee, Austin C. Via, Aldo Heredia, Daniel A. Adjei, Michael D. Bartlett

**Affiliations:** ^1^ Mechanical Engineering Soft Materials and Structures Lab Virginia Tech Blacksburg VA 24061 USA; ^2^ Electrical Engineering Virginia Tech Blacksburg VA 24061 USA; ^3^ Macromolecules Innovation Institute Virginia Tech Blacksburg VA 24061 USA

**Keywords:** bio‐inspired, biomimetics, octopus‐inspired, switchable adhesion, underwater adhesion

## Abstract

Adhesives that excel in wet or underwater environments are critical for applications ranging from healthcare and underwater robotics to infrastructure repair. However, achieving strong attachment and controlled release on difficult substrates, such as those that are curved, rough, or located in diverse fluid environments, remains a major challenge. Here, an octopus‐inspired adhesive with strong attachment and rapid release in challenging underwater environments is presented. Inspired by the octopus's infundibulum structure, a compliant, curved stalk, and an active deformable membrane for multi‐surface adhesion are utilized. The stalk's curved shape enhances conformal contact on large‐scale curvatures and increases contact stress for adaptability to small‐scale roughness. These synergistic mechanisms improve contact across multiple length scales, resulting in switching ratios of over 1000 within ≈30 ms with consistent attachment strength of over 60 kPa on diverse surfaces and conditions. These adhesives are demonstrated through the robust attachment and precise manipulation of rough underwater objects.

## Graphical abstract

An octopus‐inspired switchable adhesive (OSA) is presented that strongly attaches to and rapidly releases from challenging objects and surfaces on‐demand in underwater environments. By leveraging stalk architecture to enhance conformal contact, OSAs attach over multiple cycles and over extended time for the underwater manipulation of irregular objects from light and fragile to heavy and rigid.

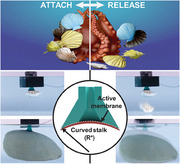

## 1 Introduction

Robust adhesives that strongly bond while also controllably releasing in wet or underwater environments are required in diverse fields including bio‐medical applications,^[^ ^1^, ^2^ ^]^ wearable electronics,^[^ ^3^, ^4^, ^5^ ^]^ and soft robotics.^[^ ^6^, ^7^, ^8^ ^]^ However, strong underwater attachment is a significant challenge due to the existence of a layer of water at the interface.^[^ ^9^, ^10^, ^11^, ^12^, ^13^ ^]^ Attachment becomes even more complicated in “real‐world” environments which often display irregular surfaces and challenging conditions.^[^ ^14^, ^15^ ^]^ For example, rough or curved surfaces reduce the contact area between the attachment device and substrate which can limit adhesion strength.^[^ ^16^, ^17^, ^18^, ^19^, ^20^ ^]^ The surface energy of the materials in contact is another factor that affects interaction forces, where lower surface energy materials form weaker attraction forces.^[^ ^21^, ^22^, ^23^ ^]^ Furthermore, the types of fluid at the interface affect the interaction between adhesive and surface.^[^ ^24^ ^]^ For example, seawater, which includes various ions, can result in lower adhesion compared to deionized water due to Debye screening effects.^[^ ^25^ ^]^ Overall, these diverse factors can reduce attachment strength and ultimately decrease the effectiveness of underwater attachment and controlled release in relevant conditions.

To achieve robust attachment in wet environments, several different methods have been adopted. For example, hydrogels, which have the ability to absorb water at the interface, and contain functional groups such as amino, carboxyl, or hydroxyl groups can create strong adhesion.^[^ ^25^, ^26^, ^27^ ^]^ Additionally, chemical modification of adhesives can promote strong bonding to the substrate through chemical reactions in a wet environment.^[^ ^28^, ^29^, ^30^ ^]^ Although hydrogels and chemical modification each provide robust adhesion performance in wet environments, these approaches are often focused on permanent adhesives and are not readily released. To create a strong yet reversible attachment, switchable adhesives can strongly attach while switching to a low force for release through a prescribed stimulus.^[^ ^31^, ^32^, ^33^, ^34^ ^]^ In wet or submerged environments, switchable attachment can be achieved through several different mechanisms, which includes capillary forces, hydrostatics (i.e., suction), hydrodynamics, and/or surface adhesion.^[^ ^34^, ^35^, ^36^ ^]^ To achieve these attachment characteristics in wet or submerged environments, organisms such as the octopus provide inspiration with their outstanding ability to manipulate underwater objects.^[^ ^37^, ^38^, ^39^ ^]^ For underwater environments, octopus‐inspired adhesives have been shown to attach and detach from wet or submerged objects.^[^ ^35^, ^40^, ^41^, ^42^, ^43^, ^44^ ^]^ This attachment is often attributed to the ability to generate interfacial pressure,^[^ ^45^, ^46^, ^47^ ^]^ which is the difference between external pressure and pressure within the attachment structure. However, strong attachment and controlled release on non‐ideal substrates is difficult,^[^ ^48^ ^]^ due to an inability to form, maintain, and rapidly control interfacial pressure in synthetic attachment structures. Therefore, synthetic materials with strong attachment and rapid, controlled release across diverse surfaces remains a major challenge.^[^ ^49^ ^]^

**Figure 1:**
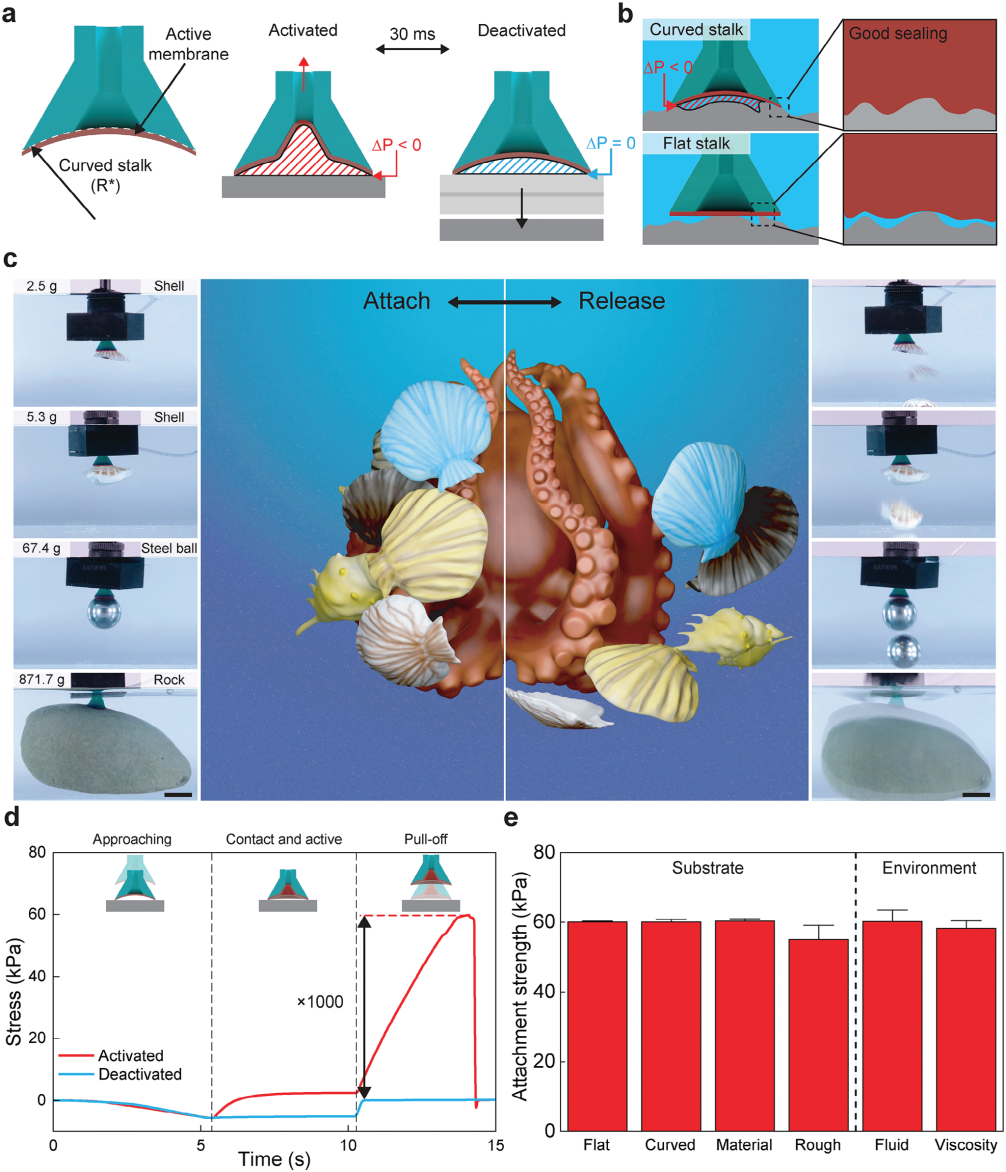
Octopus‐inspired switchable adhesives. a) Schematic of an octopus‐inspired switchable adhesive cross‐section and actuation of the membrane in the activated and deactivated state. b) Effect of curvature on the ability to seal interfacial pressure on an irregular surface. c) Attach‐and‐release octopus schematic and underwater manipulation demonstration of the octopus‐inspired switchable adhesive on irregular surfaces (scale bar = 15 mm). d) Stress versus time for an activated and deactivated OSA during a pull‐off test underwater. e) Underwater attachment strength on various substrates and environmental conditions. Bar represents the mean value ± s.d. All data are acquired with R* = 25 mm.

Here, we present an octopus‐inspired adhesive that strongly attaches and rapidly releases in diverse underwater environments and conditions. Inspired by the infundibulum structure of the octopus, we create a compliant, curved stalk coupled with an active, deformable membrane that changes shape for multi‐surface adhesion (**Figure** **1**a). The stalk curvature enhances attachment across diverse surfaces by increasing conformal contact on large‐scale curvature and by increasing contact stress around the stalk perimeter for adaptability to small‐scale roughness. These synergistic mechanisms increase contact across several length scales which increases interfacial pressure for robust underwater attachment and controllable release (Figure 1b). Our octopus‐inspired switchable adhesive (OSA) strongly attaches yet rapidly releases (≈30 ms, Figure S2, Supporting Information) complex objects from lightweight shells (2.5 g) to large rocks (872 g) that have coupled roughness and curvature across multiple scales (Figure 1c; Video S1, Supporting Information). This switching is achieved by deflecting the membrane with pneumatic‐pressure to achieve attachment switching ratios up to 1000× from the activated to deactivated state (Figure 1d). The attachment strength is consistently high (≈ 60 kPa) across various conditions, including substrate material, substrate curvature and roughness, testing fluid type, and testing fluid viscosity (Figure 1e). This approach provides a mechanism for strong yet rapid release to diverse underwater objects and surfaces even in challenging and difficult environments.

## 2 Results

### 2.1 Characterization of the Octopus‐Inspired Switchable Adhesive

The octopus‐inspired switchable adhesive (OSA) consists of an active membrane (Red) and an architectured stalk (Green) (Figure 1a). The stalk has a curvature (1/R* > 0, where R* is the radius of curvature) on the contacting surface for enhanced control of contact formation and an external stalk angle for enhanced compliance. The diameter of the OSA is 15 mm (a real image of R* = 15 mm can be found in Figure S1, Supporting Information). The active membrane is actuated to attach to and release from objects by applying a pressure differential in the stalk cavity through a pneumatic system, where Δ*P* = *P*_*input*_ − *P*_*ambient*_. Application of negative pressure (Δ*P* < 0) activates adhesion to grasp an object while neutral pressure (Δ*P* = 0) releases an object. The membrane is highly deformable, which effectively communicates the pressure differential in the stalk cavity to the interface to generate interfacial pressure for attachment. For the case of active control of underwater adhesion, octopus‐inspired adhesives have leveraged several different actuation mechanisms such as magnetic, thermal, electrostatic, and fluidic.^[^ ^49^ ^]^ We utilize a fluidic/pneumatic system as it enables us to digitally program and control the adhesive from the activated state to the deactivated state and at intermediate values (depending on the magnitude of pressure applied to the membrane) for precise control of attachment strength (Figure S3, Supporting Information). In contrast to film‐terminated attachment structures, such as mushroom shaped pillars,^[^ ^45^ ^]^ or flat top contacts,^[^ ^47^ ^]^ our architecture enables preload over the entire contact structure while maintaining compliance at the edge for reliable contact formation. One of the primary contributions to attachment with OSAs is the utilization of suction through the generation of interfacial pressure between the adhesive and the substrate. This pressure differential at the interface is a result of the increase in volume when the membrane is actuated, which reduces the interfacial pressure and creates a hydrostatic stress which can aid in attachment. However, one key challenge is the ability to both generate a stable interfacial pressure for attachment but then rapidly and controllably reduce interfacial pressure for release. The active membrane in the OSA enables the pressure differential to be precisely controlled, which overcomes challenges with controlled release in passive systems.^[^ ^49^ ^]^ However, irregular surfaces can disrupt the ability to generate interfacial pressure. This can include surface roughness and curvature which can make it difficult to maintain robust sealing for underwater attachment. Therefore, the ability to create robust sealing on irregular surface is critical to successful underwater manipulation.

**Figure 2:**
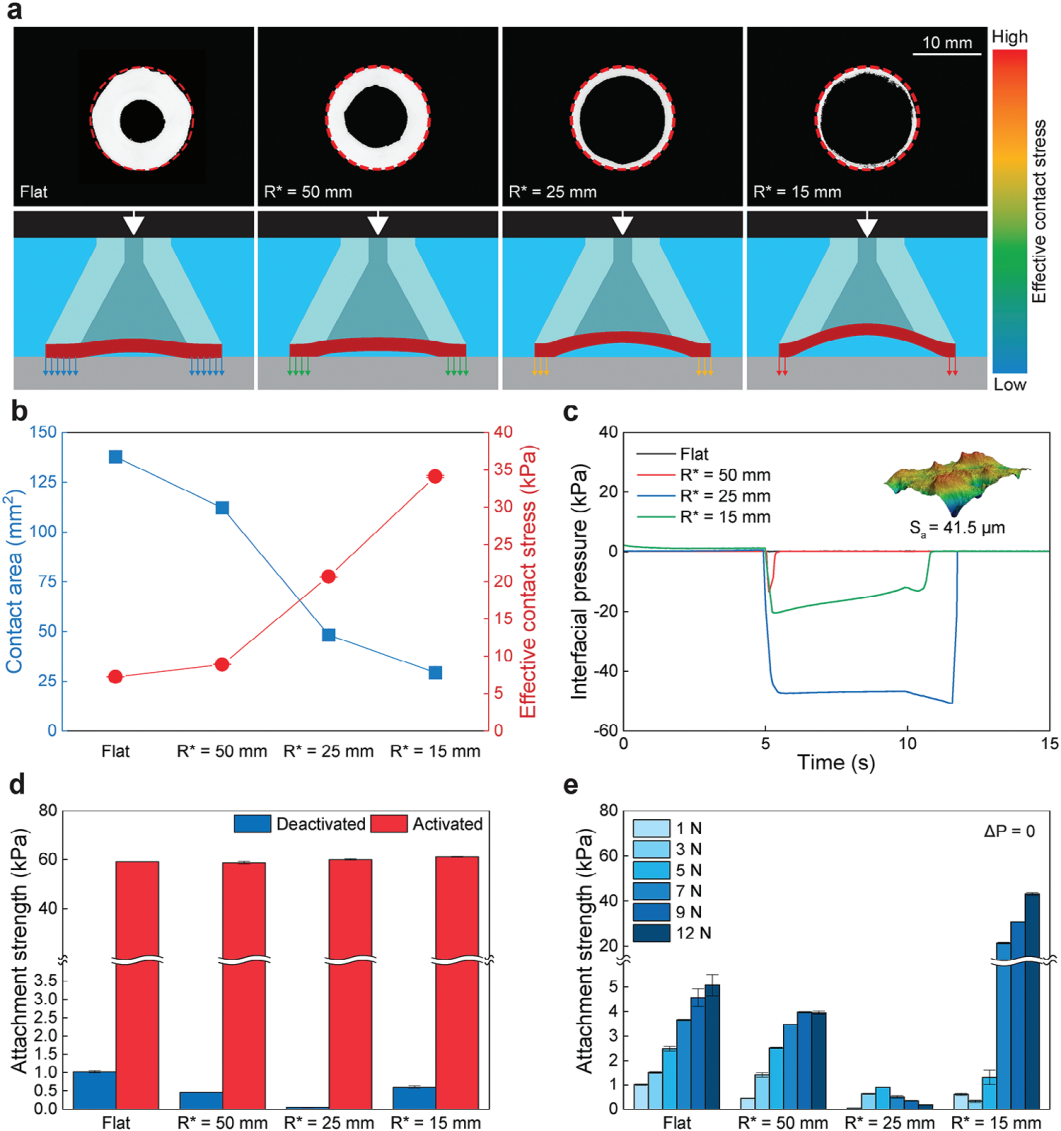
Attachment mechanism of the OSA. a) Contact area photographs with a 1 N preload for different stalk curvatures and corresponding schematics showing the effective stress depending on stalk curvature. b) Measured underwater contact area with a 1 N preload and calculated effective contact stress. c) Interfacial pressure during an attachment test on a rough, dry surface (S_*a*_ = 41.5 µm, as shown in the inset). d) Underwater attachment strength in the deactivated (blue) and activated (red) states for different stalk curvature with a 1 N preload. e) Preload dependence of underwater attachment strength in the deactivated state for different stalk curvatures. Data in (b, d, e) represent the mean value ± s.d. (*n* = 3).

To investigate the influence of stalk contact curvature on underwater attachment and release, we systematically quantified its impact on several key parameters: effective contact area, sealing performance assessed through interfacial pressure measurements, adhesion switchability, and preload dependent attachment behavior. This is assessed with a stalk with a flat contacting surface and three curved stalks with increasing curvature of R* = 50, 25, and 15 mm. First, the contact area is observed during underwater attachment with a constant preload. This is performed through a custom adhesion setup with a substrate plate that has a frustrated total internal reflection (FTIR) attachment to enhance contrast^[^ ^50^ ^]^ (**Figure** **2**a). Image analysis reveals that the effective contact area decreases with increasing curvature of the stalk at a given 1 N preload. The effective contact stress (i.e., force/effective contact area) therefore increases with increasing curvature as seen in Figure 2b, which generates locally high contact stresses which can enhance the sealing of interfacial pressure.

The effect of enhanced contact stress on sealing performance was evaluated by measuring the interfacial pressure during a pull‐off test on a rough surface (*S*_*a*_ = 41.5 µm). Here a pressure sensor is integrated into the substrate to measure the pressure in a dry environment and the membrane was actuated with Δ*P* ≤ −85 kPa. Based on the experimental data, flat and R* = 50 mm exhibit poor sealing as indicated by zero interfacial pressure or unstable or leaky sealing respectively (Figure 2c). However, R* = 25 and 15 mm show high interfacial pressure due to the enhanced conformability on the rough surface. However, the two samples show different interfacial pressure and sealing characteristics. R* = 25 mm has a greater interfacial pressure without noticeable decay of pressure, while R* = 15 mm has smaller maximum interfacial pressure with a gradual decay of pressure over time. Even though R* = 15 mm has the highest contact stress, the small contact area is susceptible to defects which can cause leaks. Additionally, increased curvature reduces the volume of the internal chamber, which can reduce the interfacial pressure. Meanwhile, the R* = 25 mm has high contact stress and enough contact area to be more defect tolerant while also having a larger internal volume. This prevents leaking while allowing the generation of high interfacial pressure.

Stalk curvature also impacts attachment switchability on smooth surfaces. In the activated state, attachment strength is constant ≈60 kPa for the flat and three curved stalks (Figure 2d). In the deactivated state, adhesion decreases with increasing stalk curvature as the contact area between the adhesive and substrate decreases (Figure 2d). However, an increase is observed for R* = 15 mm OSA which forms negative interfacial pressure when deformed.^[^ ^51^ ^]^

To further understand the release characteristics of the OSA in the deactivated (neutral) state, the attachment strength as a function of preload is evaluated. The lowest attachment strength and the smallest increase in attachment strength as preload increases occurs in the R* = 25 mm contact, where the strength difference between low and high preloads is 0.8 kPa. The R* = 25 mm sample is curved enough to maintain low contact area without large changes in volume during the preload process, providing minimal increases in attachment strength in the deactivated state and resulting in the highest switching ratio. However, flat, R* = 50 mm, and R* = 15 mm show an increase in attachment strength as preload increases, where the difference of attachment strength between the lowest and highest preloads are 4.0, 3.5, and 43.0 kPa, respectively (Figure 2e). Increased attachment strength under higher preload is attributed to greater contact at the interface for the flat and R* = 50 mm samples. The dramatic increase in the R* = 15 mm attachment strength at high preloads is attributed to the deformation of the stalk, where similar to a conventional suction cup, suction is generated due to the volume change at the interface during loading. Therefore, on a smooth flat surface stalk curvature does not impact the attachment strength which maintains ≈60 kPa in the activated state, but does influence the deactivated state. Taken together, this influences the adhesion switching ratio, where R* = 25 mm shows the highest switching ratio due to the balance of high strength with a low release strength in the deactivated state (See Figure S4, Supporting Information for switching ratio as a function of preload). For this reason, we will use R* = 25 mm for the remaining sections.

### 2.2 Attachment in Challenging Underwater Conditions

Environmental factors such as the substrate and liquid medium can limit the effectiveness of underwater adhesives.^[^ ^16^, ^17^, ^25^, ^52^ ^]^ To evaluate the performance of the OSA on different substrates, attachment strength is measured as a function of surface roughness, substrate material, and substrate curvature. Performance in different environments is then evaluated as a function of medium type and medium viscosity.

**Figure 3:**
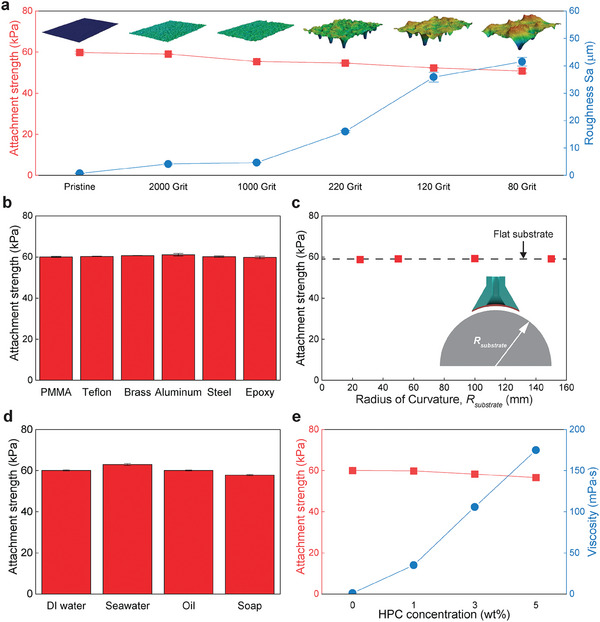
Robust attachment in diverse underwater conditions. a) Underwater attachment strength on rough surfaces (*S*_*a*_: Arithmetical mean height). b) Underwater attachment strength on diverse substrates. c) Underwater attachment strength versus surface curvature. d) Underwater attachment strength in various fluid mediums. e) Underwater attachment strength in viscous fluids. All data are acquired with R* = 25 mm. Data in (a–e) represent the mean value ± s.d. (*n* = 3).

Typically, the attachment strength of soft materials decreases dramatically as surface roughness increases.^[^ ^14^ ^]^ This is evaluated with the OSA by measuring the attachment strength on underwater surfaces molded off sandpaper with different roughness (reported in Grit). We found generally consistent attachment strengths as the roughness of the surface increases (**Figure** **3**a). For example, attachment strength reduces from 59.7 kPa on a smooth/pristine surface (Arithmetical mean height, *S*_*a*_ = 0.7 µm) to 50.8 kPa on the roughest 80 Grit surface (*S*_*a*_ = 41.5 µm). This modest decrease in strength is notable as the roughness of 80 Grit is ≈59× higher compared to the pristine surface. Figure S5 (Supporting Information) shows that stalk curvature improves the conformability of the OSA to rough surfaces. Stalk curvature creates both a greater contact area and a higher rate of percolated contact area, where the percolated contact area provides robust sealing. This provides reliable and consistent attachment strength over a wide range of surface roughnesses (Figure S6, Supporting Information). Furthermore, the OSA is capable of attaching to a wide range of underwater substrate materials. The attachment strength was found to be consistently ≈60 kPa on plastic substrates (PMMA and epoxy), low surface energy substrates (Teflon), and metallic surfaces like brass, aluminum, and steel as shown in Figure 3b. Additionally, the curved stalk geometry allows for higher tolerance on curved surfaces. We found that attachment strength was consistent over different values of the radius of curvature from 25 to 150 mm as shown in Figure 3c. This is in contrast to a notable decrease in attachment strength for OSA without stalk curvature (Figure S7, Supporting Information).

Fluid at the interface can reduce the interaction between the substrate and attachment element. To evaluate the reliability of OSA in diverse fluids, attachment strength was evaluated in various fluid types including ion‐rich fluids such as seawater simulant and organic fluids such as vegetable oil. The OSA shows a uniform attachment strength of ≈60 kPa regardless of the medium type (Figure 3d). This includes DI water, a sea‐water simulant, oil, and soapy water, showing the ability to strongly attach and release in diverse fluid conditions.

Furthermore, the effect of medium viscosity on attachment strength was evaluated by modifying the viscosity of water with different concentrations of Hydroxylpropyl cellulose (HPC). Here, as HPC concentration increases, the water viscosity increases. We find that attachment strength stays consistent with a modest drop from 60.0 to 56.6 kPa, even though viscosity increases from 1.0 *mPa* · *s* (pristine) to 175.0 *mPa* · *s* (HPC 5 wt%) (Figure 3d).

Taken together, these results show that the range of surfaces and medium conditions for underwater attachment is notably increased by utilizing an active membrane on a soft, curved stalk. This allows for control of attachment and releases while maintaining the ability to reliably seal on non‐ideal surfaces. This highlights the importance of achieving conformal contact across diverse length scales for robust underwater attachment.

### 2.3 Demonstration of the Octopus‐Inspired Adhesive

**Figure 4:**
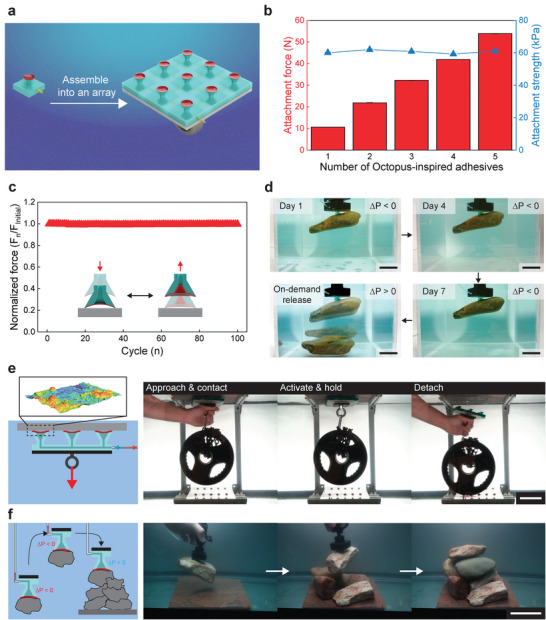
Octopus‐inspired adhesive with switchable and scalable strength. a) Scalable attachment strength through the number of OSAs (N). b) Underwater attachment force and underwater attachment strength of OSAs arrays. c) Repeatability of OSAs over 100 cycles underwater. d) Reliability of OSAs for long‐term underwater attachment (Scale bar = 40 mm). e) OSA can hold a heavy weight on a rough, underwater surface (Scale bar = 100 mm). f) OSA can attach to and controllable release underwater rocks to create a controlled assembly where the rocks have flat, curved, and rough surface features (Scale bar = 100 mm). All demonstrations are done with R* = 25 mm. Data in (b) represent represent the mean value ± s.d. (*n* = 3).

The strong, on‐demand attachment and release of OSAs to diverse underwater objects makes them compelling candidates for underwater attachment and precise manipulation. To further their utility to support larger loads, we create individual adhesive modules and assemble them into arrays as shown in **Figure** **4**a. We find that as the number of attachment elements increases from 1 to 5 the attachment force increases linearly, while the attachment strength stays constant (Figure 4b). This result shows good scalability in attachment force (See Figure S8, Supporting Information for attachment strength as a function of OSAs). In addition to scalability, the OSAs show reliable attachment over multiple cycles and over extended time. As seen in Figure 4c, the normalized attachment force stays constant over 100 cycles as the OSAs are loaded to maximum capacity. Additionally, the OSA can attach to and hold irregular‐shaped objects over an extended duration. This is demonstrated with a rock (452 g) that was held for over 7 days underwater and then released on‐demand when desired (Figure 4d). By utilizing the modularized OSA elements arranged in a 3 × 3 array, a 4.5 kg weight plate is attached on a rough surface (*S*_*a*_ = 16.0 µm) underwater (Figure 4e). Attachment and detachment of the OSAs are rapidly achieved repeatedly on‐demand Video S2, Supporting Information).

In addition to high underwater holding capacity, the OSAs can also carefully manipulate objects underwater. This capability is demonstrated by constructing an underwater cairn, a carefully constructed pile of underwater rocks. Here the rocks have various sizes, shapes, and surface roughness. First, a rock is picked up underwater by an OSA, is transported to a substrate, and is then carefully placed and released. By doing this over and over, a pile of rocks is constructed (Figure 4f; Video S3, Supporting Information). The rocks have to be precisely placed to maintain structural stability, demonstrating the ability to strongly attach to a rough, curved rock, carefully manipulate the rock into a specific position, and then controllably release the rock. These types of manipulations are performed by an octopus as they arrange objects around their den,^[^ ^53^ ^]^ and this demonstration highlights the ability of the OSA to precisely manipulate difficult underwater objects. Furthermore, the precise control of underwater attachment and release enables the damage‐free manipulation of light and fragile objects. We demonstrate this capability through the successful underwater manipulation of fragile hydrogel balls (Video S4, Supporting Information).

## 3 Discussion

This work demonstrates an octopus‐inspired adhesive with strong attachment and rapid release in challenging underwater environments. By tuning stalk curvature at the contacting surface coupled with an active membrane, we show how contact stress can be utilized to systematically control interfacial pressure. This is especially important when the substrate has roughness, curvature, or a combination of both, where the curved stalk shape enhances conformal contact on large‐scale curvatures and small‐scale roughness. This allows our OSAs to rapidly activate interfacial pressure, hold it for strong attachment, and then release it rapidly to controllable release objects on diverse surfaces and conditions. This ability is enabling for underwater manipulation tasks, where we show through the ability to achieve strong attachment and precisely manipulate irregular objects underwater. This work points to the importance of contact geometry for successful underwater attachment and release. This can guide future work on synthetic underwater adhesives, where additional contact geometries such as stalk architecture and membrane shape or tuning actuation schemes may further control attachment. It also provides insight into the contract geometry of natural octopus suckers, which typically show curvature, where future studies could quantify attachment architecture to build a better understanding of the diversity of contacting shapes. Overall, these results and designs can advance underwater and wet attachment which can be useful for diverse applications including robotic manipulation and healthcare.

## 4 Experimental Section

### Octopus‐Inspired Adhesive Fabrication

The adhesive consists of a stalk and a membrane fabricated with a silicone‐based elastomer (Dow Corning Sylgard 184). The stalk was cast and cured 10:1 PDMS (*E* = 1.6 MPa)^[^ ^54^ ^]^ in a 3D printed mold (B9R‐2‐Black photopolymer resin, B9 Creations, LLC). The active membrane was fabricated by casting 25:1 PDMS (*E* = 0.4 MPa)^[^ ^54^ ^]^ on a glass plate using a thin film applicator (ZUA 2000; Zehntner Testing Instruments) and cured at 80° C for 1 h in a convection oven. PDMS films and stalks were treated with oxygen plasma (300 mTorr, 5 min) and were then attached with a thin layer of 10:1 PDMS at the interface. The adhesive was then cured in a convection oven at 80° C for 30 min. The diameter of the OSA is 15 mm at the contacting surface, the total height is 10.5 mm, the stalk has a 30° external angle and a 3 mm stalk thickness. The angled stalk starts after a 3 mm long initial cylindrical section (with a 6.6 mm diameter) that connects to the OSA base. The stalk has an internal curvature (1/R* > 0, where R* is the radius of curvature) on the contacting surface which varies from flat to R* = 50, 25, and 15 mm.

### Fabrication of Substrate with Rough Surface

Substrates with different surface roughness were fabricated by molding Epoxy (J‐B Weld) in an elastomeric negative mold with sandpaper imprint. Sand paper was first cut into a circular shape using a laser cutter (Universal laser system) and attached to the center of a positive mold composed of staked acrylic plates. Then, Ecoflex 00‐30 (Smooth‐on) was poured into the positive mold and cured in a convection oven at 80° C for 1 h, creating compliant negative molds with a sandpaper imprint. Epoxy was then poured into the negative elastomeric mold. Epoxy is cured for 1 h at room temperature and cured in a convection oven at 80° C for 24 h to fabricate the rigid substrates with rough surfaces.

### Fabrication of Curved Substrate

Substrates with radius of curvatures (*R*_*substrate*_) of 25, 50, 100, and 150 mm and flat were used for attachment characterization. The curved surfaces were fabricated with a resin‐based 3D printer (Anycubic Photon D2). Once manufactured, the surfaces of the curved substrate were polished with 800 grit sandpaper and then a finer 2000 grit sandpaper.

### Adhesion Characterization

Adhesion was characterized by a custom setup with a pneumatic control system (Proportion‐Air) to control membrane actuation. Manipulation of pressure states depended on the stage of the test by triggering the pneumatic system with an Instron 5944 mechanical tester (Instron Mechanical Testing Systems). Attachment experiments of the OSAs consist of three stages. First, the OSA approaches the substrate with a constant speed of 1 mm s^−1^ until it reaches a target compression preload of 1 N (unless otherwise noted). Second, pressure was applied to the OSA and held for 5 s to ensure the complete application of pressure. All experiments where the OSA was activated used Δ*P* ≤ −85 kPa unless otherwise noted and Δ*P* ≤ 0 kPa for the deactivated state. Then, the OSA was pulled until complete delamination. The attachment strength in all cases was calculated from the projected area of contact (i.e., the area of the top of the OSA surface which had a diameter of 15 mm, not the actual area of contact), which provided a lower bound for the attachment strength.

### Contact Area Analysis

Contact area during attachment experiments was recorded through a 45° tilted mirror under the testing substrate. A LED strip mounted on the side of the substrate was the light source for the frustrated total internal reflection (FTIR) as shown in Figure S9 (Supporting Information). Images of the contact area were processed and analyzed using ImageJ (version 1.53K) Image Analysis Software.

## Conflict of Interest

The authors declare no conflict of interests.

## Author Contributions

C.L. and M.D.B. conceived the idea. C.L., A.V., A.H., and D.A. prepared adhesives and performed experiments. C.L. and M.D.B. analyzed the results. C.L. and M.D.B. wrote the paper. M.D.B supervised the study.

## Supporting information

Supporting Information

Supporting Video 1

Supporting Video 2

Supporting Video 3

Supporting Video 4

## Data Availability

The data that support the findings of this study are available from the corresponding author upon reasonable request.
