# Supplementary material for: Octopus‐Inspired Adhesives with Switchable Attachment to Challenging Underwater Surfaces
Source: Adv Sci (Weinh). 2024 Oct 9;12(1):2407588. doi: 10.1002/advs.202407588 (PMC11714156; doi:10.1002/advs.202407588)
Supplement: Supplementary file 1 — Supporting Information [file ADVS-12-2407588-s002.pdf]

ADVANCED SCIENCE
Open Access

Supporting Information

for *Adv. Sci.*, DOI 10.1002/advs.202407588

Octopus-Inspired Adhesives with Switchable Attachment to Challenging Underwater Surfaces

*Chanhong Lee, Austin C. Via, Aldo Heredia, Daniel A. Adjei and Michael D. Bartlett**

Supplementary Information

Octopus-inspired adhesives with switchable attachment to challenging underwater surfaces

Chanhong Lee[1], Austin C. Via[1], Aldo Heredia[1], Daniel A. Adjei[2] and Michael D. Bartlett[1,3*]

[1]Mechanical Engineering, Soft Materials and Structures Lab, Virginia Tech, Blacksburg, VA 24061, USA.

[2]Electrical Engineering, Virginia Tech, Blacksburg, VA 24061, USA.

[3]Macromolecules Innovation Institute, Virginia Tech, Blacksburg, VA 24061, USA.

*Corresponding author email: mbartlett@vt.edu

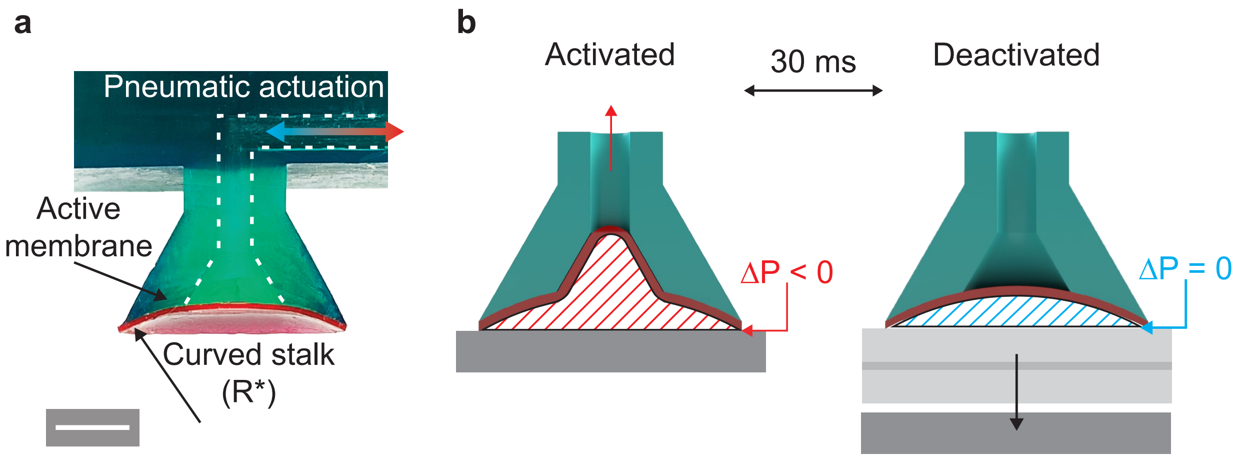

Fig. S1 Real image of octopus-inspired adhesives and the mechanism of actuation. **a**. Cross-sectional image of an octopus-inspired adhesive with $R^* = 15$ mm (scale bar = 5 mm). **b**. Pneumatic actuation of octopus-inspired adhesives in activated and deactivated states.

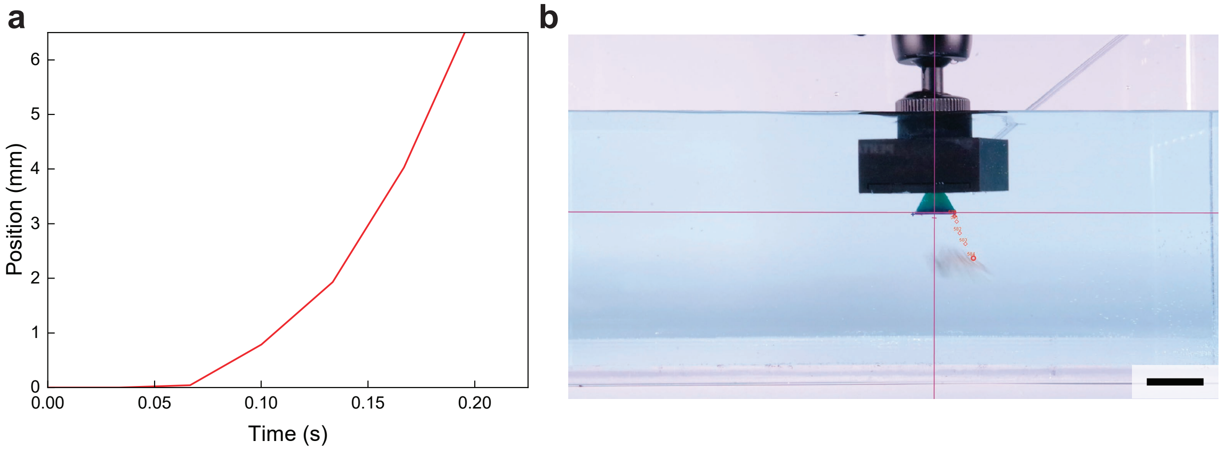

Fig. S2 Release rate measurement through image analysis. **a**. Position of object after active membrane deactivation. **b**. An example of the image analysis procedure (scale bar = 20 mm).

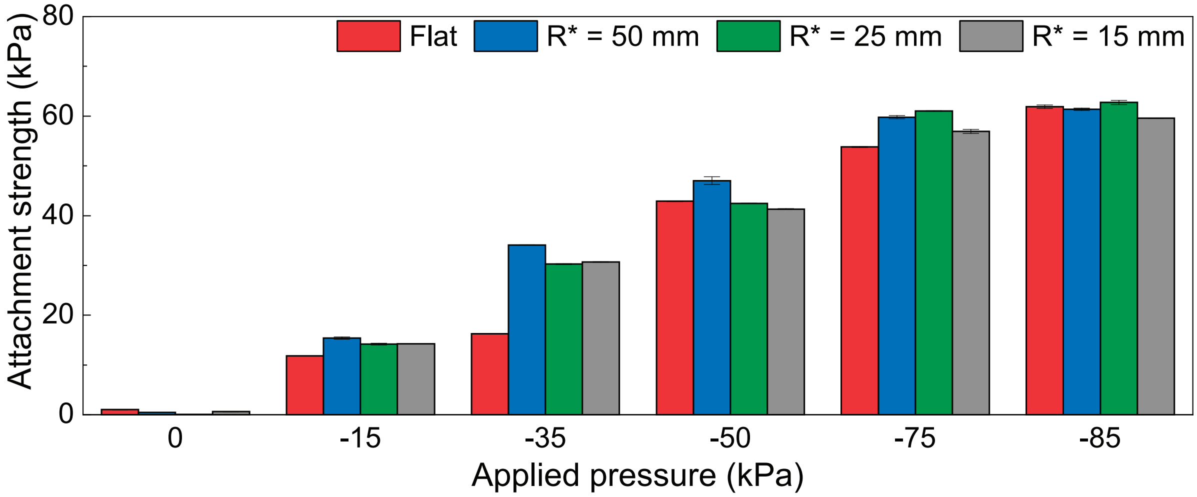

Fig. S3 Effect of applied pneumatic pressure on attachment strength.

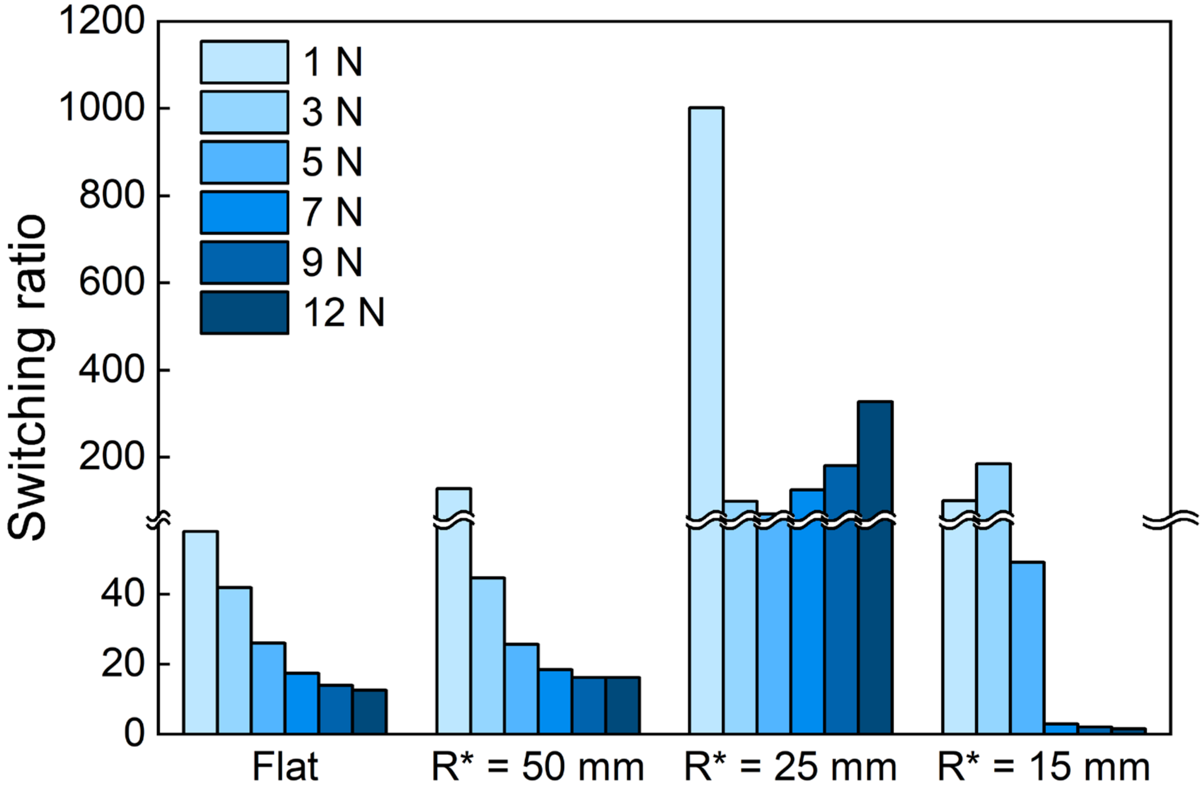

Fig. S4 Preload dependent switching ratio for different stalk curvatures.

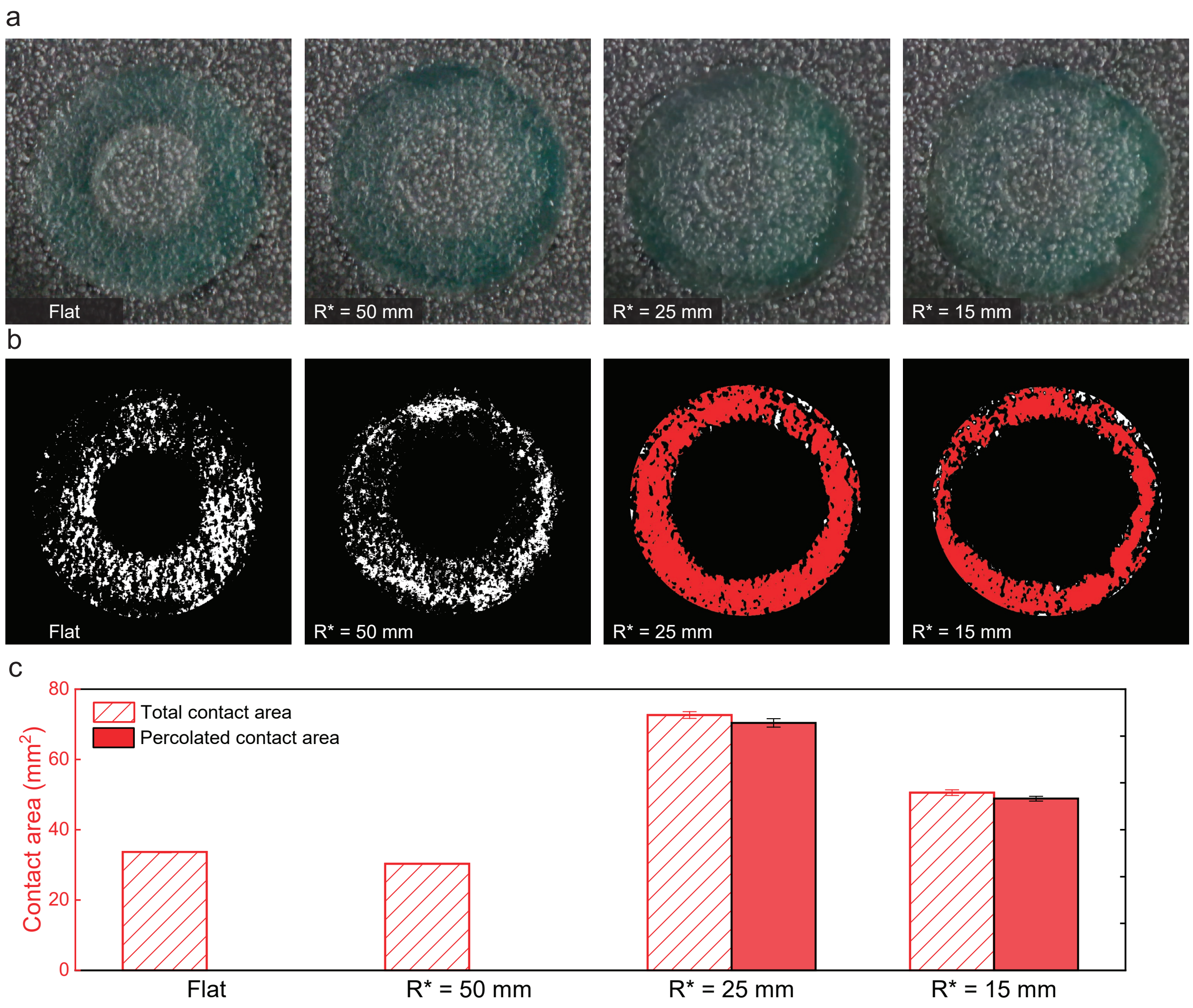

Fig. S5 OSA contact area on a rough surface for different stalk curvatures. a. Contact area images of octopus-inspired adhesives (OSAs) with different stalk curvatures on a rough surface (80 Grit) (Preload = 5 N). **b**. Processed images of the contact area. White represents the adhesive contact area on the rough surface and the red color shows the contact area of a percolated contact region. In the absence of red, there was no percolated network found in the image analysis. **c**. Plot of the contact area for different OSAs on the rough surface to compare total contact area and the percolated contact area.

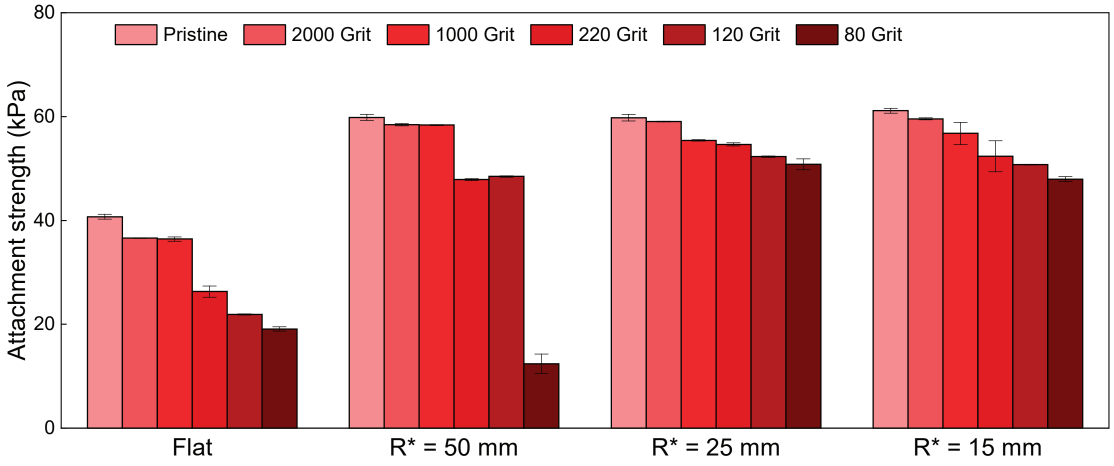

Fig. S6 Attachment strength of OSAs with different stalk curvature on various rough surfaces.

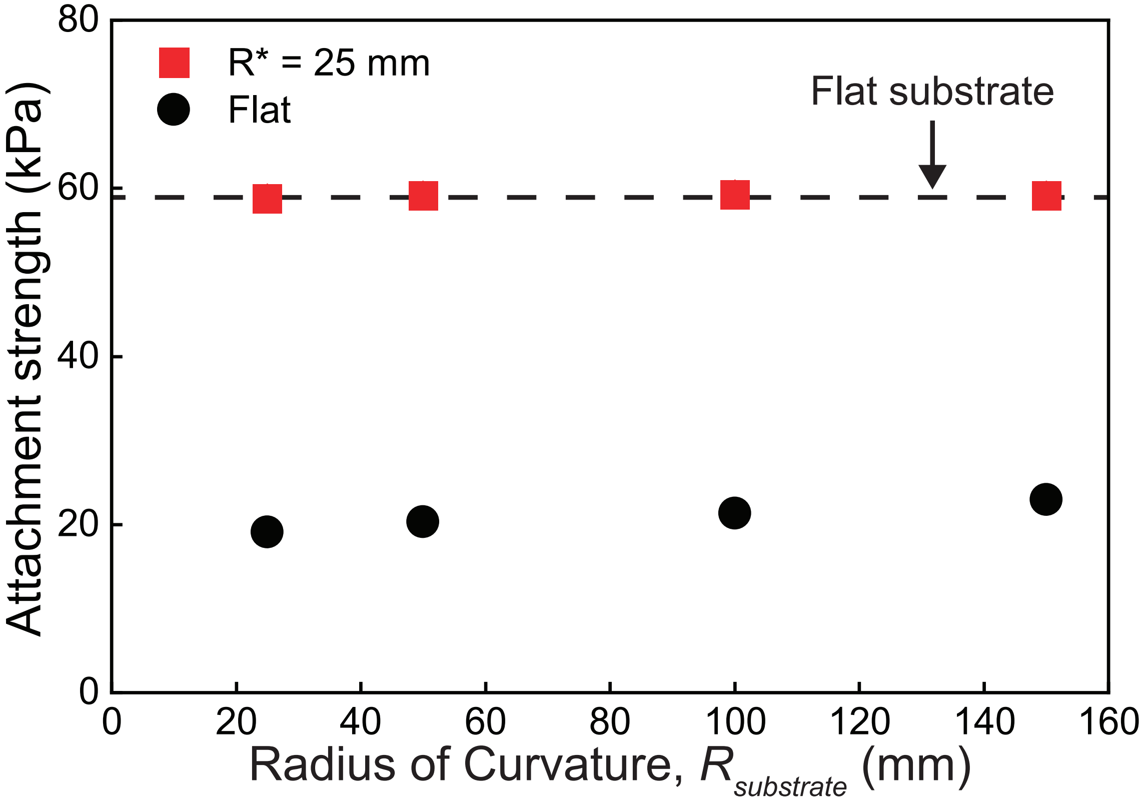

Fig. S7 Attachment strength versus radius of curvature ($R_{substrate}$). Attachment strength of an OSA with $R^* = 25$ mm and a flat OSA on a curved, underwater surface.

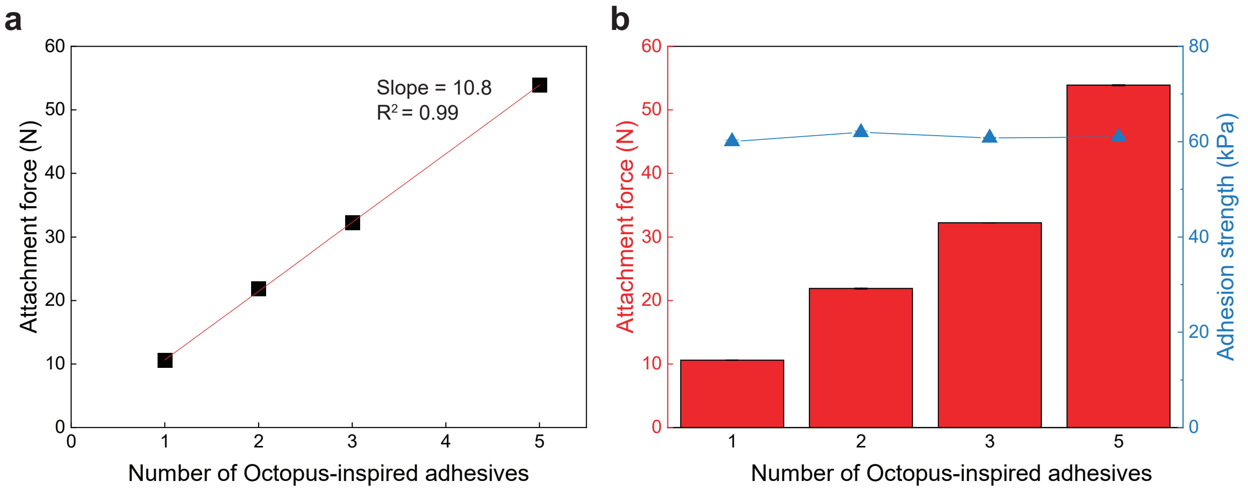

Fig. S8 Scaling OSA attachment strength through OSA arrays. **a**. Linear fit for attachment force vs number of OSAs ($R^* = 25$ mm). **b**. Attachment force and Attachment strength (force normalized by the adhesive area) as a function of the number of octopus-inspired adhesives. Data in **a and b** represent the mean value ± s.d. ($n = 3$).

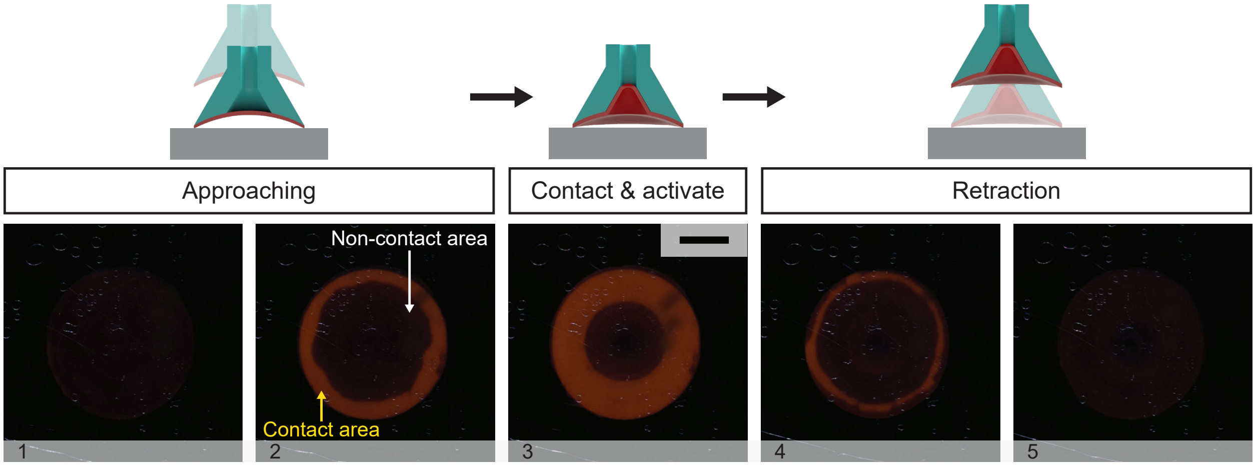

Fig. S9 Experimental contact area images during attachment measurements. An image sequence showing the contact area observed through the FTIR effect during an attachment test (scale bar = 5 mm).

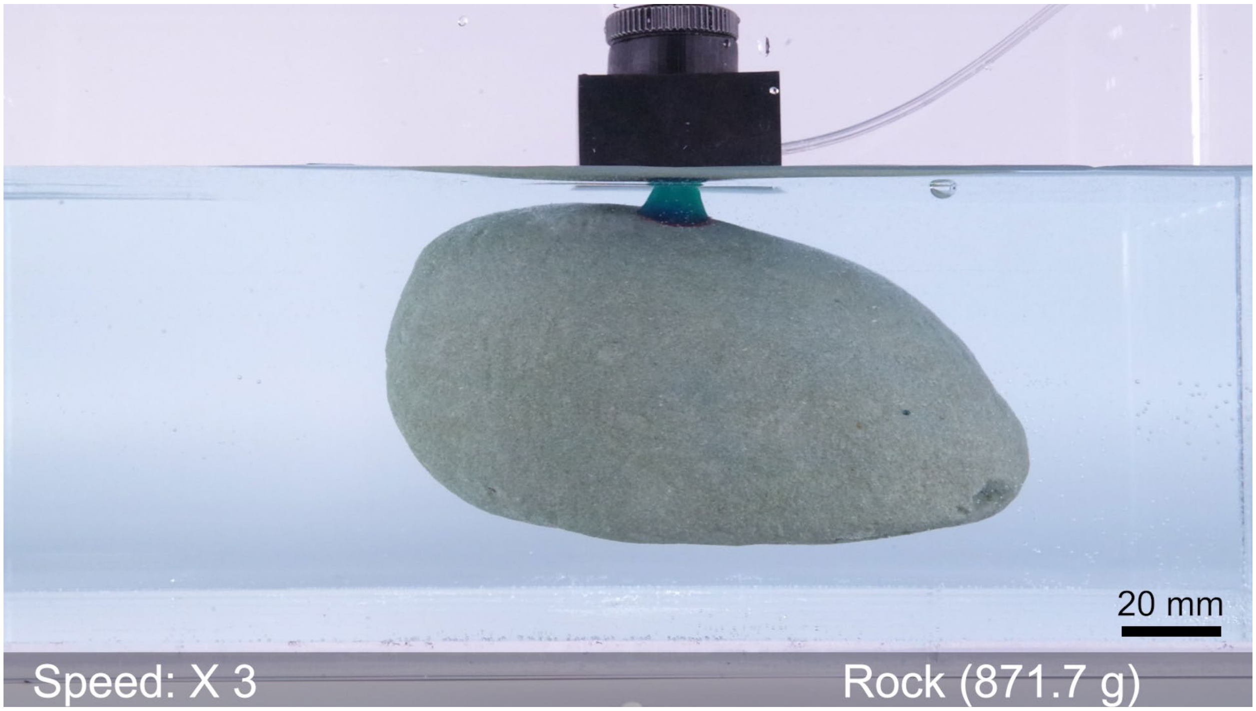

Supplementary Video S1: Attaching and releasing to underwater objects with different sizes and complex shapes.

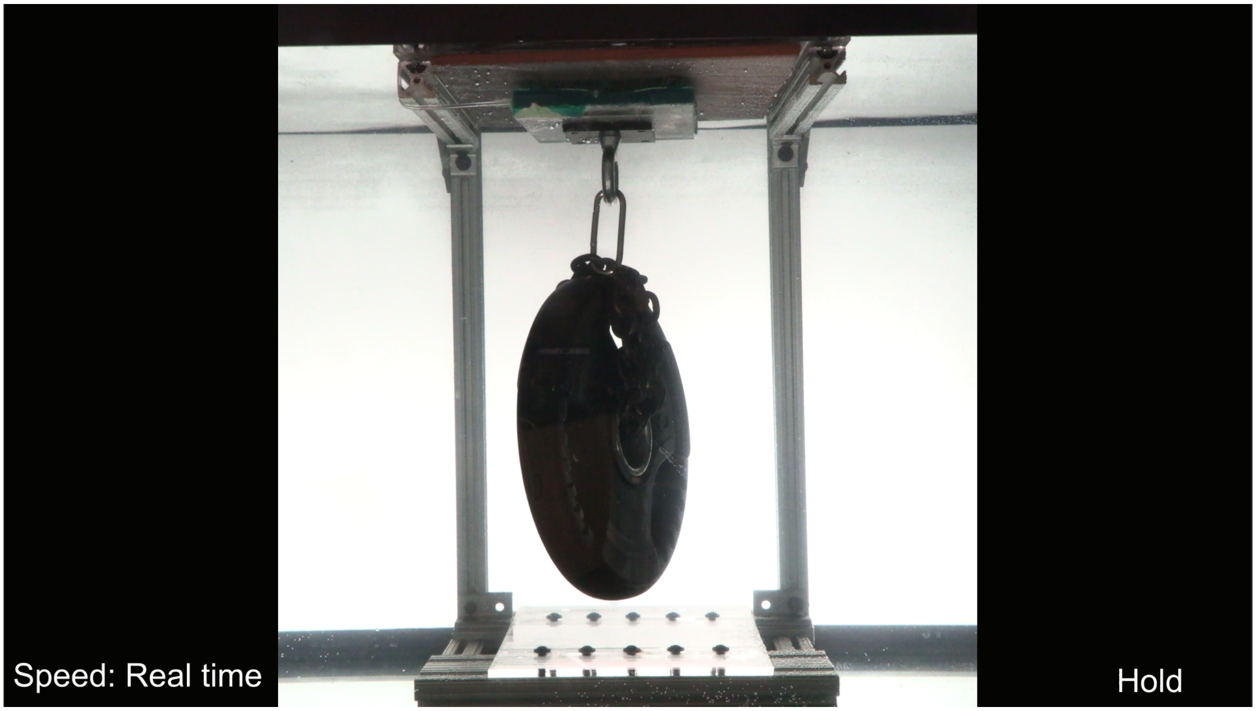

Supplementary Video S2: Strong and switchable attachment strength of an octopus-inspired adhesive on a rough, underwater surface.

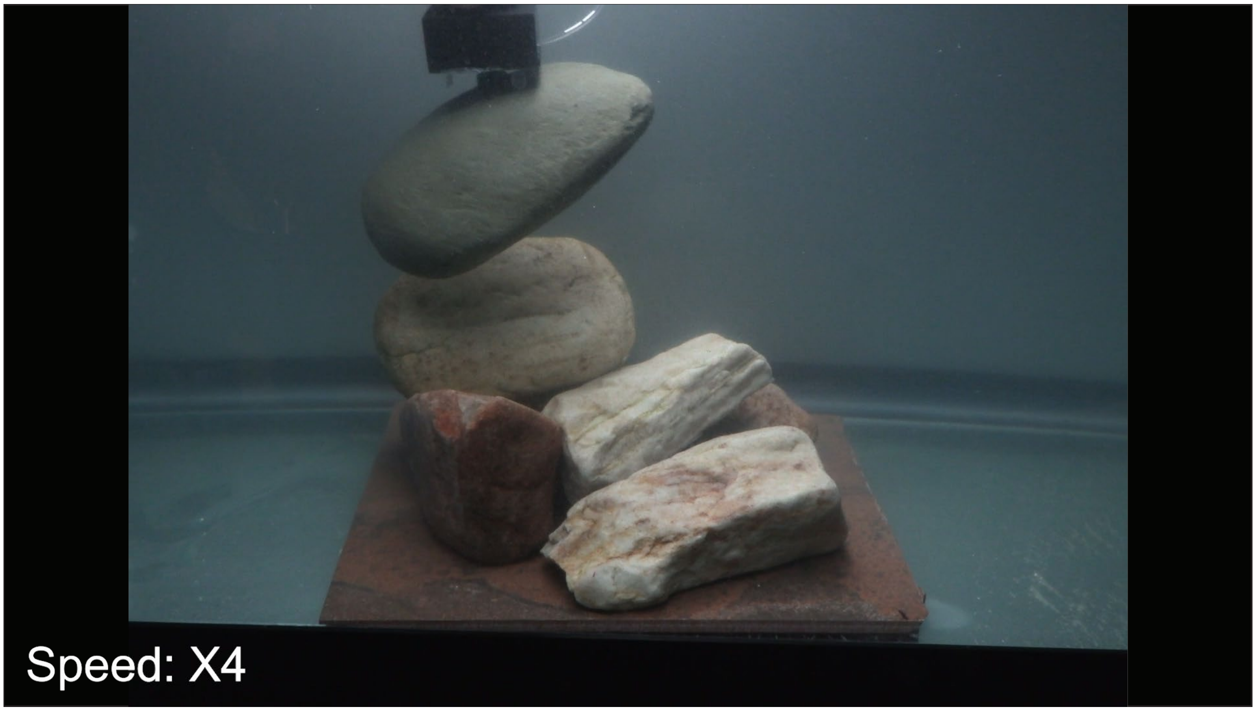

Supplementary Video S3: Precise manipulation of complex underwater objects with an octopus-inspired adhesive to construct a rock pile.

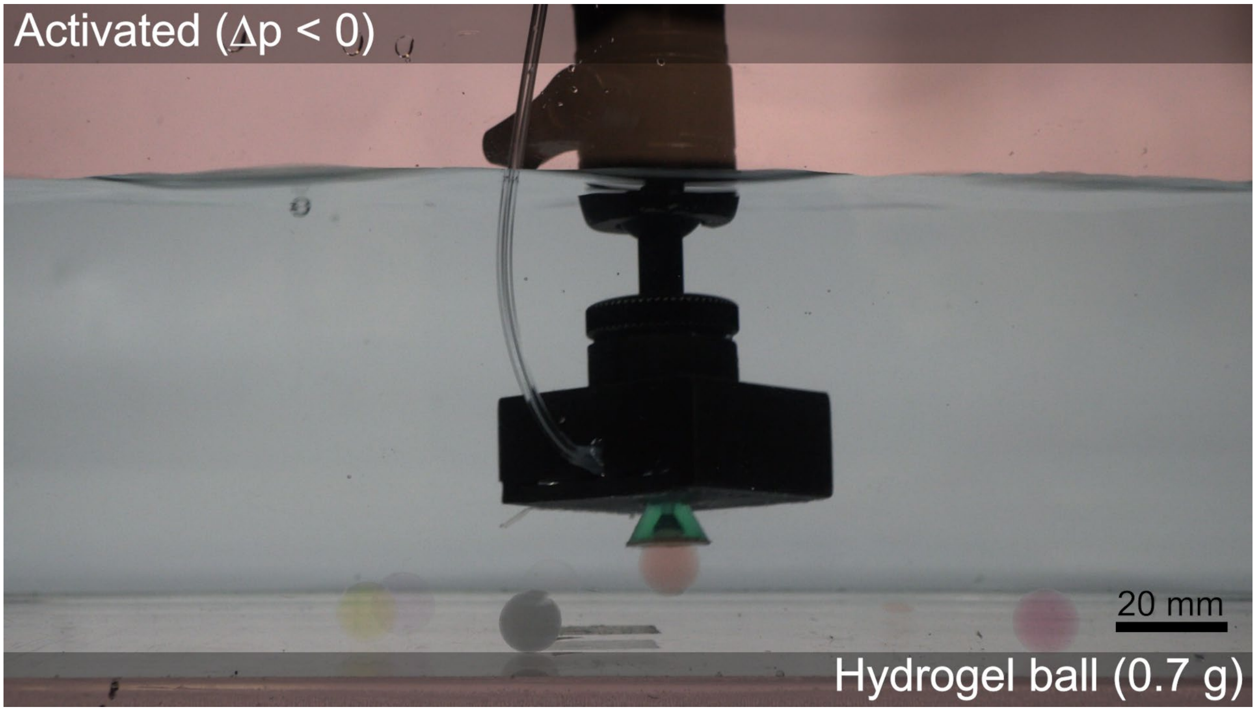

Supplementary Video S4: Underwater manipulation of a light and fragile hydrogel ball.
